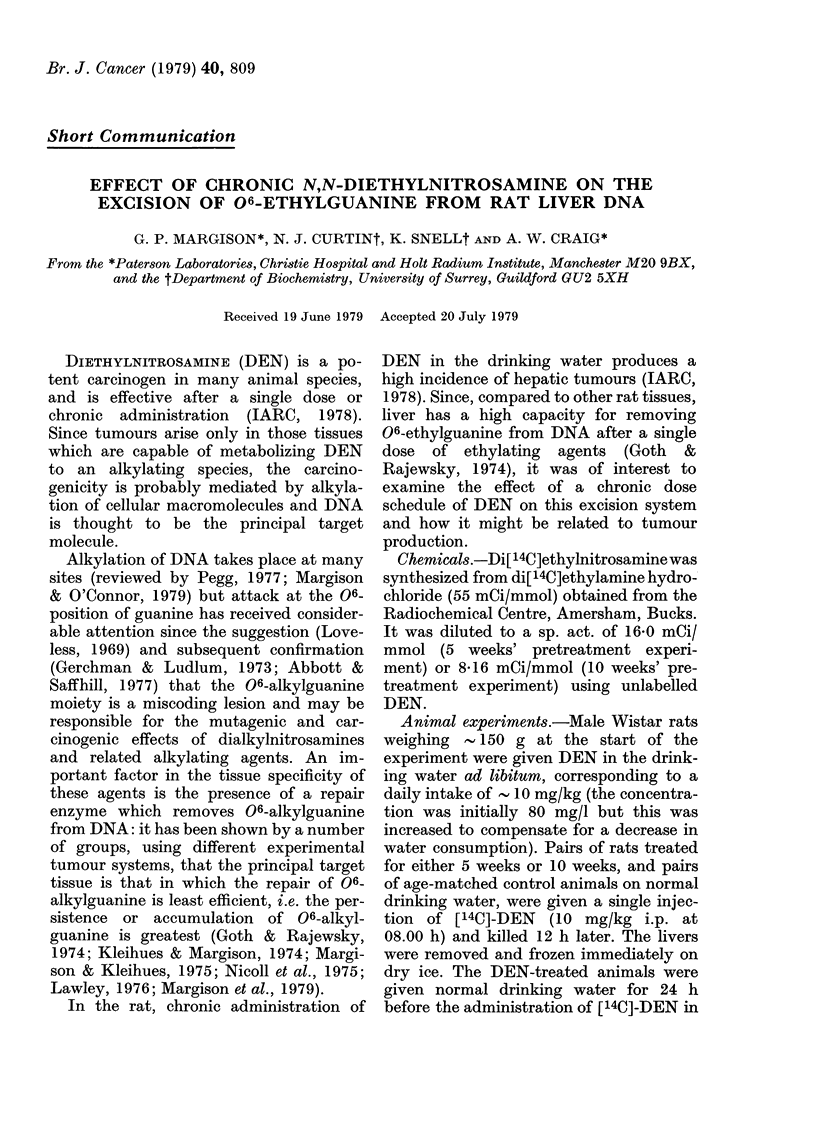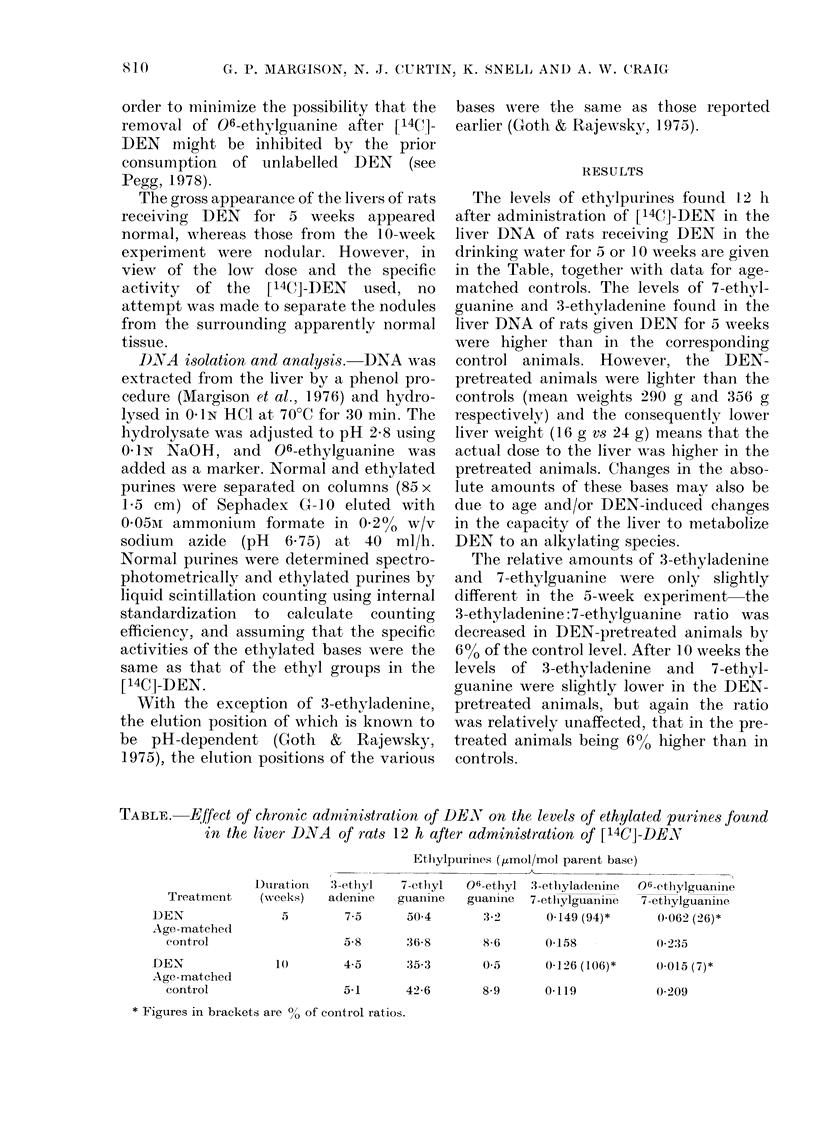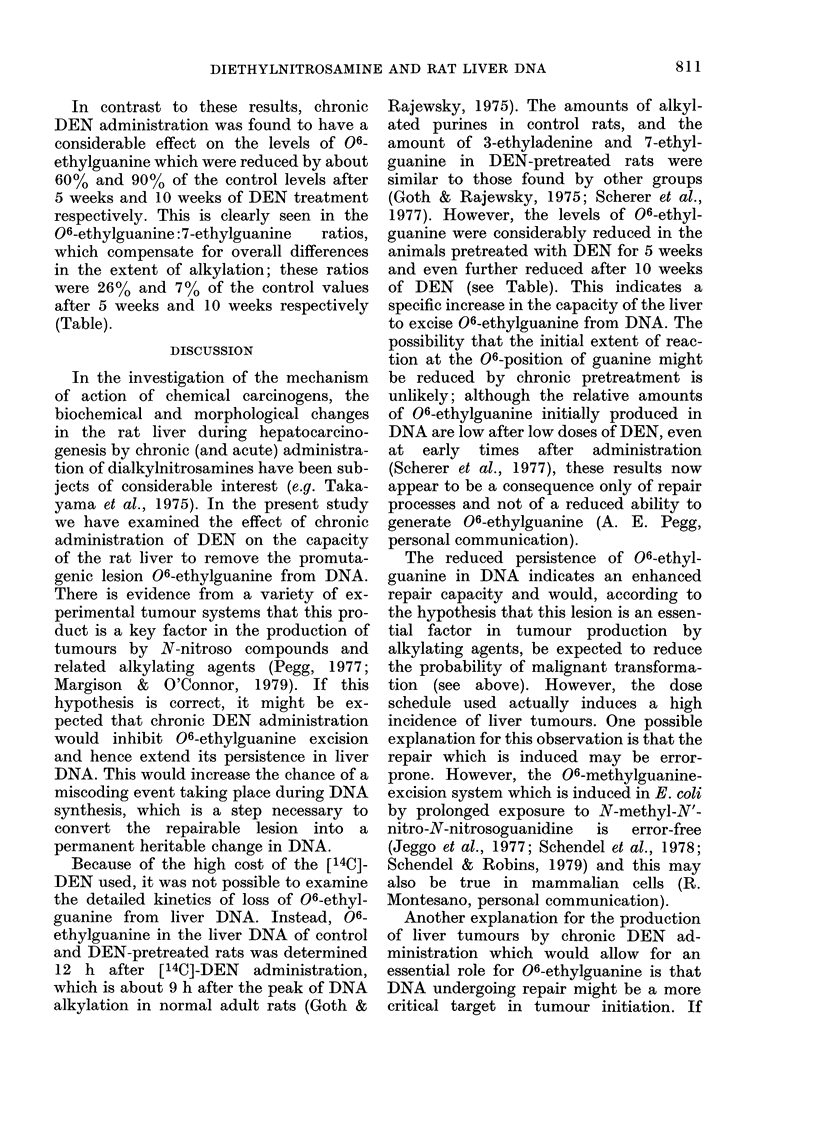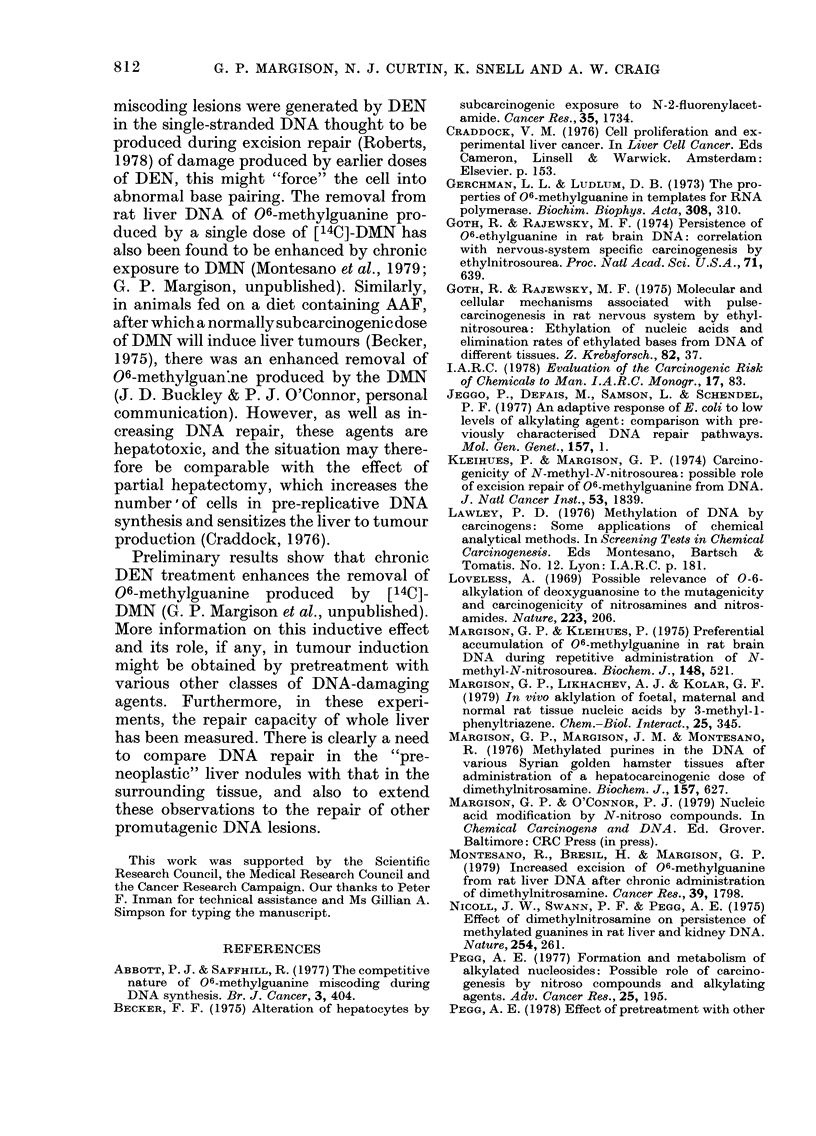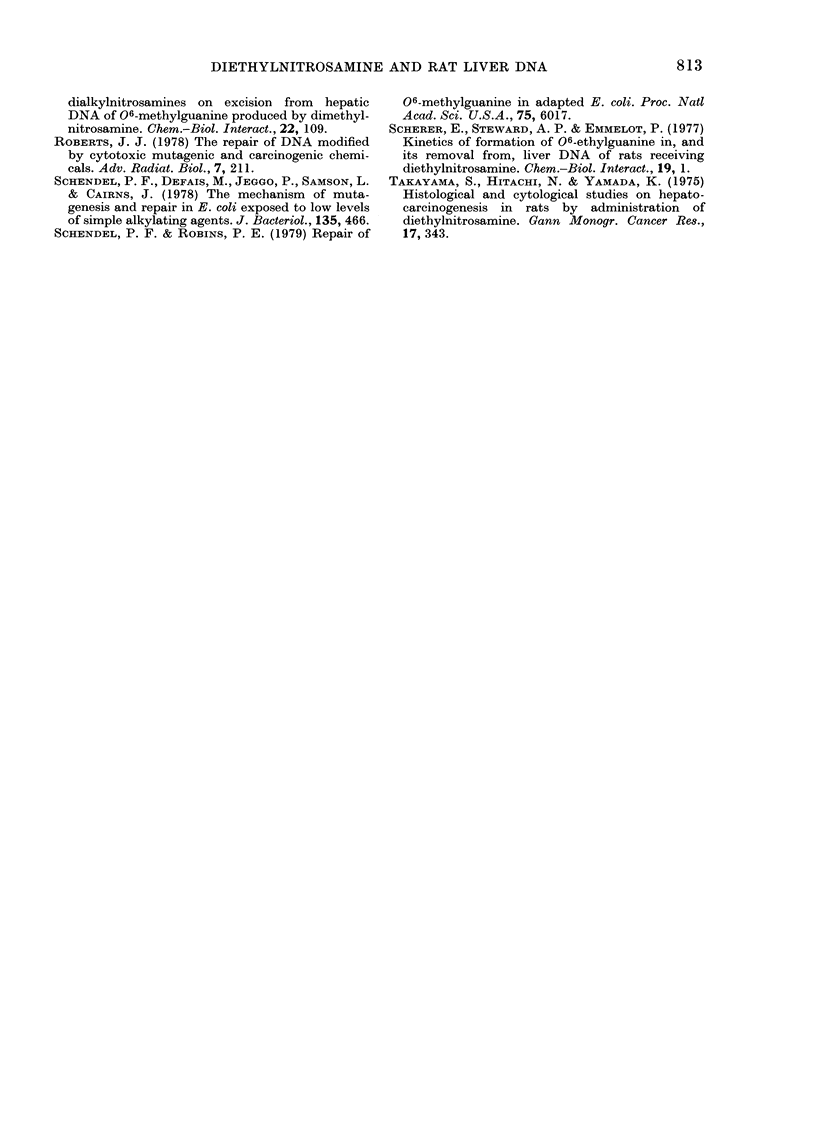# Effect of chronic N,N-diethylnitrosamine on the excision of O6-ethylguanine from rat liver DNA.

**DOI:** 10.1038/bjc.1979.266

**Published:** 1979-11

**Authors:** G. P. Margison, N. J. Curtin, K. Snell, A. W. Craig


Br. J. Cancer (1979) 40, 809

### Short Communication

### EFFECT OF CHRONIC N,N-DIETHYLNITROSAMINE ON THE EXCISION OF $O^6$-ETHYLGUANINE FROM RAT LIVER DNA

G. P. MARGISON*, N. J. CURTIN†, K. SNELL† AND A. W. CRAIG*

*From the *Paterson Laboratories, Christie Hospital and Holt Radium Institute, Manchester M20 9BX, and the †Department of Biochemistry, University of Surrey, Guildford GU2 5XH*

Received 19 June 1979 Accepted 20 July 1979

DIETHYLNITROSAMINE (DEN) is a potent carcinogen in many animal species, and is effective after a single dose or chronic administration (IARC, 1978). Since tumours arise only in those tissues which are capable of metabolizing DEN to an alkylating species, the carcinogenicity is probably mediated by alkylation of cellular macromolecules and DNA is thought to be the principal target molecule.

Alkylation of DNA takes place at many sites (reviewed by Pegg, 1977; Margison & O'Connor, 1979) but attack at the $O^6$-position of guanine has received considerable attention since the suggestion (Loveless, 1969) and subsequent confirmation (Gerchman & Ludlum, 1973; Abbott & Saffhill, 1977) that the $O^6$-alkylguanine moiety is a miscoding lesion and may be responsible for the mutagenic and carcinogenic effects of dialkylnitrosamines and related alkylating agents. An important factor in the tissue specificity of these agents is the presence of a repair enzyme which removes $O^6$-alkylguanine from DNA: it has been shown by a number of groups, using different experimental tumour systems, that the principal target tissue is that in which the repair of $O^6$-alkylguanine is least efficient, *i.e.* the persistence or accumulation of $O^6$-alkylguanine is greatest (Goth & Rajewsky, 1974; Kleihues & Margison, 1974; Margison & Kleihues, 1975; Nicoll *et al.*, 1975; Lawley, 1976; Margison *et al.*, 1979).

In the rat, chronic administration of DEN in the drinking water produces a high incidence of hepatic tumours (IARC, 1978). Since, compared to other rat tissues, liver has a high capacity for removing $O^6$-ethylguanine from DNA after a single dose of ethylating agents (Goth & Rajewsky, 1974), it was of interest to examine the effect of a chronic dose schedule of DEN on this excision system and how it might be related to tumour production.

*Chemicals.*—Di[$^{14}$C]ethylnitrosamine was synthesized from di[$^{14}$C]ethylamine hydrochloride (55 mCi/mmol) obtained from the Radiochemical Centre, Amersham, Bucks. It was diluted to a sp. act. of 16·0 mCi/mmol (5 weeks' pretreatment experiment) or 8·16 mCi/mmol (10 weeks' pretreatment experiment) using unlabelled DEN.

*Animal experiments.*—Male Wistar rats weighing ~150 g at the start of the experiment were given DEN in the drinking water *ad libitum*, corresponding to a daily intake of ~10 mg/kg (the concentration was initially 80 mg/l but this was increased to compensate for a decrease in water consumption). Pairs of rats treated for either 5 weeks or 10 weeks, and pairs of age-matched control animals on normal drinking water, were given a single injection of [$^{14}$C]-DEN (10 mg/kg i.p. at 08.00 h) and killed 12 h later. The livers were removed and frozen immediately on dry ice. The DEN-treated animals were given normal drinking water for 24 h before the administration of [$^{14}$C]-DEN in order to minimize the possibility that the removal of $O^6$-ethylguanine after [$^{14}$C]-DEN might be inhibited by the prior consumption of unlabelled DEN (see Pegg, 1978).

The gross appearance of the livers of rats receiving DEN for 5 weeks appeared normal, whereas those from the 10-week experiment were nodular. However, in view of the low dose and the specific activity of the [$^{14}$C]-DEN used, no attempt was made to separate the nodules from the surrounding apparently normal tissue.

*DNA isolation and analysis.*—DNA was extracted from the liver by a phenol procedure (Margison *et al.*, 1976) and hydrolysed in 0·1N HCl at 70°C for 30 min. The hydrolysate was adjusted to pH 2·8 using 0·1N NaOH, and $O^6$-ethylguanine was added as a marker. Normal and ethylated purines were separated on columns (85 × 1·5 cm) of Sephadex G-10 eluted with 0·05M ammonium formate in 0·2% w/v sodium azide (pH 6·75) at 40 ml/h. Normal purines were determined spectrophotometrically and ethylated purines by liquid scintillation counting using internal standardization to calculate counting efficiency, and assuming that the specific activities of the ethylated bases were the same as that of the ethyl groups in the [$^{14}$C]-DEN.

With the exception of 3-ethyladenine, the elution position of which is known to be pH-dependent (Goth & Rajewsky, 1975), the elution positions of the various bases were the same as those reported earlier (Goth & Rajewsky, 1975).

### RESULTS

The levels of ethylpurines found 12 h after administration of [$^{14}$C]-DEN in the liver DNA of rats receiving DEN in the drinking water for 5 or 10 weeks are given in the Table, together with data for age-matched controls. The levels of 7-ethylguanine and 3-ethyladenine found in the liver DNA of rats given DEN for 5 weeks were higher than in the corresponding control animals. However, the DEN-pretreated animals were lighter than the controls (mean weights 290 g and 356 g respectively) and the consequently lower liver weight (16 g *vs* 24 g) means that the actual dose to the liver was higher in the pretreated animals. Changes in the absolute amounts of these bases may also be due to age and/or DEN-induced changes in the capacity of the liver to metabolize DEN to an alkylating species.

The relative amounts of 3-ethyladenine and 7-ethylguanine were only slightly different in the 5-week experiment—the 3-ethyladenine:7-ethylguanine ratio was decreased in DEN-pretreated animals by 6% of the control level. After 10 weeks the levels of 3-ethyladenine and 7-ethylguanine were slightly lower in the DEN-pretreated animals, but again the ratio was relatively unaffected, that in the pretreated animals being 6% higher than in controls.

TABLE.—*Effect of chronic administration of DEN on the levels of ethylated purines found in the liver DNA of rats 12 h after administration of [$^{14}$C]-DEN*

| Treatment | Duration (weeks) | Ethylpurines (μmol/mol parent base) 3-ethyl adenine | 7-ethyl guanine | $O^6$-ethyl guanine | 3-ethyladenine / 7-ethylguanine | $O^6$-ethylguanine / 7-ethylguanine |
|---|---|---|---|---|---|---|
| DEN | 5 | 7·5 | 50·4 | 3·2 | 0·149 (94)* | 0·062 (26)* |
| Age-matched control | | 5·8 | 36·8 | 8·6 | 0·158 | 0·235 |
| DEN | 10 | 4·5 | 35·3 | 0·5 | 0·126 (106)* | 0·015 (7)* |
| Age-matched control | | 5·1 | 42·6 | 8·9 | 0·119 | 0·209 |

* Figures in brackets are % of control ratios.

In contrast to these results, chronic DEN administration was found to have a considerable effect on the levels of $O^6$-ethylguanine which were reduced by about 60% and 90% of the control levels after 5 weeks and 10 weeks of DEN treatment respectively. This is clearly seen in the $O^6$-ethylguanine:7-ethylguanine ratios, which compensate for overall differences in the extent of alkylation; these ratios were 26% and 7% of the control values after 5 weeks and 10 weeks respectively (Table).

### DISCUSSION

In the investigation of the mechanism of action of chemical carcinogens, the biochemical and morphological changes in the rat liver during hepatocarcinogenesis by chronic (and acute) administration of dialkylnitrosamines have been subjects of considerable interest (*e.g.* Takayama *et al.*, 1975). In the present study we have examined the effect of chronic administration of DEN on the capacity of the rat liver to remove the promutagenic lesion $O^6$-ethylguanine from DNA. There is evidence from a variety of experimental tumour systems that this product is a key factor in the production of tumours by *N*-nitroso compounds and related alkylating agents (Pegg, 1977; Margison & O'Connor, 1979). If this hypothesis is correct, it might be expected that chronic DEN administration would inhibit $O^6$-ethylguanine excision and hence extend its persistence in liver DNA. This would increase the chance of a miscoding event taking place during DNA synthesis, which is a step necessary to convert the repairable lesion into a permanent heritable change in DNA.

Because of the high cost of the [$^{14}$C]-DEN used, it was not possible to examine the detailed kinetics of loss of $O^6$-ethylguanine from liver DNA. Instead, $O^6$-ethylguanine in the liver DNA of control and DEN-pretreated rats was determined 12 h after [$^{14}$C]-DEN administration, which is about 9 h after the peak of DNA alkylation in normal adult rats (Goth & Rajewsky, 1975). The amounts of alkylated purines in control rats, and the amount of 3-ethyladenine and 7-ethylguanine in DEN-pretreated rats were similar to those found by other groups (Goth & Rajewsky, 1975; Scherer *et al.*, 1977). However, the levels of $O^6$-ethylguanine were considerably reduced in the animals pretreated with DEN for 5 weeks and even further reduced after 10 weeks of DEN (see Table). This indicates a specific increase in the capacity of the liver to excise $O^6$-ethylguanine from DNA. The possibility that the initial extent of reaction at the $O^6$-position of guanine might be reduced by chronic pretreatment is unlikely; although the relative amounts of $O^6$-ethylguanine initially produced in DNA are low after low doses of DEN, even at early times after administration (Scherer *et al.*, 1977), these results now appear to be a consequence only of repair processes and not of a reduced ability to generate $O^6$-ethylguanine (A. E. Pegg, personal communication).

The reduced persistence of $O^6$-ethylguanine in DNA indicates an enhanced repair capacity and would, according to the hypothesis that this lesion is an essential factor in tumour production by alkylating agents, be expected to reduce the probability of malignant transformation (see above). However, the dose schedule used actually induces a high incidence of liver tumours. One possible explanation for this observation is that the repair which is induced may be error-prone. However, the $O^6$-methylguanine-excision system which is induced in *E. coli* by prolonged exposure to *N*-methyl-*N'*-nitro-*N*-nitrosoguanidine is error-free (Jeggo *et al.*, 1977; Schendel *et al.*, 1978; Schendel & Robins, 1979) and this may also be true in mammalian cells (R. Montesano, personal communication).

Another explanation for the production of liver tumours by chronic DEN administration which would allow for an essential role for $O^6$-ethylguanine is that DNA undergoing repair might be a more critical target in tumour initiation. If miscoding lesions were generated by DEN in the single-stranded DNA thought to be produced during excision repair (Roberts, 1978) of damage produced by earlier doses of DEN, this might "force" the cell into abnormal base pairing. The removal from rat liver DNA of $O^6$-methylguanine produced by a single dose of [$^{14}$C]-DMN has also been found to be enhanced by chronic exposure to DMN (Montesano *et al.*, 1979; G. P. Margison, unpublished). Similarly, in animals fed on a diet containing AAF, after which a normally subcarcinogenic dose of DMN will induce liver tumours (Becker, 1975), there was an enhanced removal of $O^6$-methylguanine produced by the DMN (J. D. Buckley & P. J. O'Connor, personal communication). However, as well as increasing DNA repair, these agents are hepatotoxic, and the situation may therefore be comparable with the effect of partial hepatectomy, which increases the number of cells in pre-replicative DNA synthesis and sensitizes the liver to tumour production (Craddock, 1976).

Preliminary results show that chronic DEN treatment enhances the removal of $O^6$-methylguanine produced by [$^{14}$C]-DMN (G. P. Margison *et al.*, unpublished). More information on this inductive effect and its role, if any, in tumour induction might be obtained by pretreatment with various other classes of DNA-damaging agents. Furthermore, in these experiments, the repair capacity of whole liver has been measured. There is clearly a need to compare DNA repair in the "preneoplastic" liver nodules with that in the surrounding tissue, and also to extend these observations to the repair of other promutagenic DNA lesions.

This work was supported by the Scientific Research Council, the Medical Research Council and the Cancer Research Campaign. Our thanks to Peter F. Inman for technical assistance and Ms Gillian A. Simpson for typing the manuscript.

REFERENCES

ABBOTT, P. J. & SAFFHILL, R. (1977) The competitive nature of $O^6$-methylguanine miscoding during DNA synthesis. *Br. J. Cancer*, **3**, 404.

BECKER, F. F. (1975) Alteration of hepatocytes by subcarcinogenic exposure to N-2-fluorenylacetamide. *Cancer Res.*, **35**, 1734.

CRADDOCK, V. M. (1976) Cell proliferation and experimental liver cancer. In *Liver Cell Cancer.* Eds Cameron, Linsell & Warwick. Amsterdam: Elsevier. p. 153.

GERCHMAN, L. L. & LUDLUM, D. B. (1973) The properties of $O^6$-methylguanine in templates for RNA polymerase. *Biochim. Biophys. Acta*, **308**, 310.

GOTH, R. & RAJEWSKY, M. F. (1974) Persistence of $O^6$-ethylguanine in rat brain DNA: correlation with nervous-system specific carcinogenesis by ethylnitrosourea. *Proc. Natl Acad. Sci. U.S.A.*, **71**, 639.

GOTH, R. & RAJEWSKY, M. F. (1975) Molecular and cellular mechanisms associated with pulse-carcinogenesis in rat nervous system by ethylnitrosourea: Ethylation of nucleic acids and elimination rates of ethylated bases from DNA of different tissues. *Z. Krebsforsch.*, **82**, 37.

I.A.R.C. (1978) *Evaluation of the Carcinogenic Risk of Chemicals to Man. I.A.R.C. Monogr.*, **17**, 83.

JEGGO, P., DEFAIS, M., SAMSON, L. & SCHENDEL, P. F. (1977) An adaptive response of *E. coli* to low levels of alkylating agent: comparison with previously characterised DNA repair pathways. *Mol. Gen. Genet.*, **157**, 1.

KLEIHUES, P. & MARGISON, G. P. (1974) Carcinogenicity of *N*-methyl-*N*-nitrosourea: possible role of excision repair of $O^6$-methylguanine from DNA. *J. Natl Cancer Inst.*, **53**, 1839.

LAWLEY, P. D. (1976) Methylation of DNA by carcinogens: Some applications of chemical analytical methods. In *Screening Tests in Chemical Carcinogenesis.* Eds Montesano, Bartsch & Tomatis. No. 12. Lyon: I.A.R.C. p. 181.

LOVELESS, A. (1969) Possible relevance of *O*-6-alkylation of deoxyguanosine to the mutagenicity and carcinogenicity of nitrosamines and nitrosamides. *Nature*, **223**, 206.

MARGISON, G. P. & KLEIHUES, P. (1975) Preferential accumulation of $O^6$-methylguanine in rat brain DNA during repetitive administration of *N*-methyl-*N*-nitrosourea. *Biochem. J.*, **148**, 521.

MARGISON, G. P., LIKHACHEV, A. J. & KOLAR, G. F. (1979) *In vivo* aklylation of foetal, maternal and normal rat tissue nucleic acids by 3-methyl-1-phenyltriazene. *Chem.-Biol. Interact.*, **25**, 345.

MARGISON, G. P., MARGISON, J. M. & MONTESANO, R. (1976) Methylated purines in the DNA of various Syrian golden hamster tissues after administration of a hepatocarcinogenic dose of dimethylnitrosamine. *Biochem. J.*, **157**, 627.

MARGISON, G. P. & O'CONNOR, P. J. (1979) Nucleic acid modification by *N*-nitroso compounds. In *Chemical Carcinogens and DNA.* Ed. Grover. Baltimore: CRC Press (in press).

MONTESANO, R., BRESIL, H. & MARGISON, G. P. (1979) Increased excision of $O^6$-methylguanine from rat liver DNA after chronic administration of dimethylnitrosamine. *Cancer Res.*, **39**, 1798.

NICOLL, J. W., SWANN, P. F. & PEGG, A. E. (1975) Effect of dimethylnitrosamine on persistence of methylated guanines in rat liver and kidney DNA. *Nature*, **254**, 261.

PEGG, A. E. (1977) Formation and metabolism of alkylated nucleosides: Possible role of carcinogenesis by nitroso compounds and alkylating agents. *Adv. Cancer Res.*, **25**, 195.

PEGG, A. E. (1978) Effect of pretreatment with other dialkylnitrosamines on excision from hepatic DNA of $O^6$-methylguanine produced by dimethylnitrosamine. *Chem.–Biol. Interact.*, **22**, 109.

ROBERTS, J. J. (1978) The repair of DNA modified by cytotoxic mutagenic and carcinogenic chemicals. *Adv. Radiat. Biol.*, **7**, 211.

SCHENDEL, P. F., DEFAIS, M., JEGGO, P., SAMSON, L. & CAIRNS, J. (1978) The mechanism of mutagenesis and repair in *E. coli* exposed to low levels of simple alkylating agents. *J. Bacteriol.*, **135**, 466.

SCHENDEL, P. F. & ROBINS, P. E. (1979) Repair of $O^6$-methylguanine in adapted *E. coli*. *Proc. Natl Acad. Sci. U.S.A.*, **75**, 6017.

SCHERER, E., STEWARD, A. P. & EMMELOT, P. (1977) Kinetics of formation of $O^6$-ethylguanine in, and its removal from, liver DNA of rats receiving diethylnitrosamine. *Chem.–Biol. Interact.*, **19**, 1.

TAKAYAMA, S., HITACHI, N. & YAMADA, K. (1975) Histological and cytological studies on hepatocarcinogenesis in rats by administration of diethylnitrosamine. *Gann Monogr. Cancer Res.*, **17**, 343.